# Psychopathological and Neurobiological Overlap Between Anorexia Nervosa and Self-Injurious Behavior: A Narrative Review and Conceptual Hypotheses

**DOI:** 10.3389/fpsyt.2022.756238

**Published:** 2022-05-11

**Authors:** Marloes Oudijn, Jara Linders, Roel Mocking, Anja Lok, Annemarie van Elburg, D. Denys

**Affiliations:** ^1^Department of Psychiatry, Amsterdam University Medical Centers (Amsterdam UMC), Academic Medical Center (AMC), University of Amsterdam, Amsterdam, Netherlands; ^2^Faculty of Social Sciences, University of Utrecht, Utrecht, Netherlands

**Keywords:** anorexia nervosa, eating disorders, non-suicidal self-injurious behavior, self-destruction, psychopathology, neurobiology

## Abstract

Empirical evidence and clinical observations suggest a strong -yet under acknowledged-link between anorexia nervosa (AN) and non-suicidal self-injurious behavior (NSSI). By reviewing the literature on the psychopathology and neurobiology of AN and NSSI, we shed light on their relationship. Both AN and NSSI are characterized by disturbances in affect regulation, dysregulation of the reward circuitry and the opioid system. By formulating a reward-centered hypothesis, we explain the overlap between AN and NSSI. We propose three approaches understanding the relationship between AN and NSSI, which integrate psychopathology and neurobiology from the perspective of self-destructiveness: (1) a nosographical approach, (2) a research domain (RDoC) approach and (3) a network analysis approach. These approaches will enhance our knowledge of the underlying neurobiological substrates and may provide groundwork for the development of new treatment options for disorders of self-destructiveness, like AN and NSSI. In conclusion, we hypothesize that self-destructiveness is a new, DSM-5-transcending concept or psychopathological entity that is reward-driven, and that both AN and NSSI could be conceptualized as disorders of self-destructiveness.

## Introduction

Empirical evidence and clinical observation suggest a strong link between eating disorders (EDs) and non-suicidal self-injurious behavior (NSSI). Up to 72% of patients with EDs also engage in NSSI, and 25–54% of patients that engage in NSSI report comorbid disordered eating (1).

In addition, NSSI and EDs share clinical risk factors and there is overlap in motivational and behavioral aspects of both disorders (2), suggesting shared underlying (neurobiological) mechanisms.

In our recent study on deep brain stimulation (DBS) as a potential new treatment option for anorexia nervosa (AN) we saw an increase in self-destructive behavior when the eating-disorder symptoms lost their rewarding properties (3). This rose the question how AN and self-destructive behavior are linked, whether there is a shared pathogenesis, and why this potential shared pathogenesis leads to different clinical expressions. On a more fundamental level, we wondered whether the self-starvation of AN-patients, which seems to be an ultimate form of self-destruction, could be considered primarily self-destructive. In this review self-destructive or self-injurious behavior is narrowed down to NSSI and suicidal intent is excluded.

As is common in psychiatric practice, therapeutic options often aim at either eating disorder pathology or self-injurious behavior (4, 5). If NSSI and EDs share etiopathogenetic mechanisms, acknowledging them may improve understanding, diagnosis and treatment efficacy. However, research is scarce and there is currently no overview on the neurobiological association between EDs and NSSI.

Although all EDs evidently show pathological eating behaviors with related cognitions and emotions, suggesting a common root, there are also significant clinical and etiopathogenic differences between EDs. Since the clinical overlap between self-destructive behavior and one particular eating disorder, namely anorexia nervosa, seems to be the most striking, and most neurobiological evidence is available for AN, we will focus this review on AN.

With this narrative review we summarize the literature on the psychopathology and neurobiology of anorexia nervosa (AN) and NSSI and shed new light on their links (6).

We propose a new conceptualization of the overlap between AN and NSSI and suggest new approaches to better characterize their relation. Thereby we aim at providing a groundwork for future research and the development of new biological treatment options for AN and NSSI, especially if they are occurring simultaneously.

## Methods and Outline

We conducted this narrative review according to the steps described by Demiris (6). To answer the above questions we first summarize the literature on the psychopathology (clinical picture and psychological theories on the etiopathogenesis) and the neurobiology of AN and NSSI separately. For this search we used various combinations of search terms regarding “Anorexia nervosa” “Self-destructive behavior,” “NSSI” AND/OR “psychopathology” AND/OR “neurobiology.” We chose relevant articles that focus on the combination of and/or overlap between the two. Because this search did not reveal many relevant hits we expanded the search with “Eating disorders” AND “Self-destructive behavior” for topics on which there was no literature specific to AN. We scanned reference lists of included articles for additional relevant literature.

First, we summarize the psychopathology and neurobiology of AN and NSSI separately. In chapter 3 we discuss the commonality between AN and NSSI on a psychopathological and neurobiological level, leading to a reward centered hypothesis on this overlap. We made the somewhat artificial distinction between psychopathology in general and neurobiology in specific for reasons of clarification from a research perspective. Finally we propose three conceptual hypotheses explaining the relationship between AN and NSSI, integrating psychopathology and neurobiology.

## Results: Psychopathology and Neurobiology of Anorexia Nervosa and Non-Suicidal Self-Injurious Behavior, Respectively

### Anorexia Nervosa

#### Psychopathology of Anorexia Nervosa

Anorexia nervosa (AN) is an eating disorder characterized by (1) an intense fear of gaining weight or becoming fat; (2) persistent behavior that interferes with weight gain, potentially leading to self-starvation and severe somatic complications; (3) and a misperception of one’s body weight or shape (and often also the seriousness of the malnutritive state of the body). There are two subtypes of AN: the restrictive subtype (AN-R), characterized by restrictive eating; and the binge eating/purging subtype (AN-BP), characterized by restrictive energy intake but also binging and/or purging (self-induced vomiting, misuse of laxatives and/or diuretics) (7).

AN typically has its onset in adolescence (8) and the female-male ratio is 4:1 (9). AN has a prevalence of 1–4% and (9, 10). With a mortality rate of 5.6% per decade (resulting from medical complications and suicide), AN has the highest mortality rate of all psychiatric disorders (9, 11).

Psychiatric comorbidity is high: comorbid psychiatric disorders are reported in over 70% of AN-patients and include affective disorders, obsessive compulsive disorder, personality disorders, and impulse control disorders (9). Studies found correlations between AN and childhood trauma and between AN and specific personality traits (like perfectionism, negative emotionality and bodily dissatisfaction (12). Despite increased knowledge of neurobiological, psychological and environmental factors that contribute to the development and maintenance of the (9, 13, 14), effective treatment options are limited, and AN takes on a chronic course in a considerable number of patients (20%) (15).

#### Neurobiology of Anorexia Nervosa

Several neurobiological factors have been investigated in AN, including neurotransmitter systems and brain network functioning (16). Changes in serotonin (5-HT) and dopamine (DA) have been most often examined.

Several studies show alterations in 5-HT-metabolites, -receptor binding potential and -activity, suggesting involvement of 5-HT function in AN (17, 18). Dopamine (DA) is related to motivation and reward, and to eating and the reinforcing value of food (19). Studies on DA metabolites and receptor density in AN suggest an altered DA functioning and disturbed reward processing in AN (19–22). Bailer et al. (23) found a positive association between DA release and anxiety in the dorsal caudate in AN patients, possibly explaining why food-related DA-release produces anxiety in AN but is considered pleasurable in healthy individuals.

Brain areas involved in eating and eating disorders can be classified in circuits that are interconnected and mutually interact with each other. Although difficult to compare, functional magnetic resonance imaging (fMRI) studies in AN show consistently altered activation in emotional, reward and cognitive brain networks as well as networks implied in interoception. In Supplementary Table 1, we summarize fMRI studies in ill and recovered AN patients according to Frank et al. and O’Hara et al. (24, 25).

In summary, the structures showing dysfunctional activation in ill and recovered AN patients are part of (1) salience and reward networks related to emotional processing and reward processing, and (2) a cortical cognitive circuit related to selective attention and planning. Together they form the cortico-striatal-limbic neurocircuit, which is implicated also in other reward related psychiatric disorders such as obsessive compulsive disorder (OCD) (26).

Several models refer to a dysfunctional reward circuit in AN [e.g., the bottom-up top-down model by Kaye et al. (27)]. Frank et al. (24) suggest that there is a conflict between the conscious motivation to restrict food in AN and a body-homeostasis driven motivation to approach food. There seems to be a reinforcing mechanism between AN behavior and anxiety. Some AN behavioral symptoms provide short time relief of anxiety and stress and are rewarding.

Based on these findings, O’Hara et al. (25) formulated a reward-based model of AN. In this model the etiopathophysiology of AN is described as an increased reward responsiveness for and habit formation of anorectic behavior. AN is thought to originate in striatal reward dysfunction, reflected by reward-based learning of stimulus-driven bottom-up processes. Illness-compatible cues become positively associated with reward, while food-related healthy cues lose their rewarding properties, and instead become punishing, a process the authors call ‘reward contamination’.

### Non-suicidal Self-Injurious Behavior

#### Psychopathology of Non-suicidal Self-Injurious Behavior

As opposed to AN, non-suicidal self-injurious behavior (NSSI) is no disorder or disease category. NSSI is defined as “any socially unaccepted behavior involving deliberate and direct destruction of bodily tissue without suicidal intent” (28–30). Examples of NSSI are skin-cutting, self-hitting, self-burning and scratching. In the DSM-III, NSSI was described as a symptom of emotional and developmental disorders like borderline personality disorder (BPD) and impulse control disorder (31). The DSM-5 has included NSSI in the category “Conditions for Further Study” (7), which, according to Cipriano et al. (32), is a first step toward recognizing NSSI as a separate disorder. One of the criteria of NSSI in the DMS-5 is that the behavior attempts to diminish inter- as well as intrapersonal psychological discomfort.

Since self-injurious behavior is often studied simultaneously with suicidal behavior, it is difficult to separate findings for NSSI from suicidality. Moreover, self-injurious behavior has been studied under various acronyms [e.g., (non-suicidal) self-injury, self-mutilation, self-harm, deliberate self-harm, parasuicidal behavior] with varying in- and exclusion criteria (with the most prominent variation being the in- or exclusion of suicidal intent), which complicates interpretation of the literature. In this review self-injurious behavior is narrowed down to NSSI and suicidal intent is excluded.

Non-suicidal self-injurious behavior has an onset in early adolescence (12–14 years) and a higher prevalence in women than in men (29, 32). According to a recent systematic review by Cipriano et al. (32), NSSI has a prevalence of 4–23% in adults and 7.5–46.5% in adolescents, with wide ranges due to differences in samples and methods. The prevalence rates of NSSI in psychiatric populations are higher: 20% in adult psychiatric populations and 40–80% in adolescent psychiatric populations (29, 33). Because of the association of NSSI with psychiatric conditions (borderline personality disorder, depressive disorder, anxiety disorders, post-traumatic stress disorder, substance abuse and eating disorders) individuals engaging in NSSI are part of a diagnostically heterogeneous population.

Accordingly, the described etiopathogenesis of NSSI is diverse. Several studies found environmental or social risk factors for NSSI, like childhood trauma, attachment problems and dysfunctional interpersonal relationships, as well as individual risk factors like emotion regulation problems (32, 34). Paivio and McCulloch (35) showed that difficulties to identify and express emotional experience appropriately (i.e.,. alexithymia) mediated the relation between childhood trauma (except sexual abuse) and NSSI.

Most studies on NSSI describe emotion regulation problems as the primary source. Typically, an increase in negative emotionality is observed before engaging in NSSI, which is reduced after NSSI, resulting in a positive rewarding experience (32). Ammerman et al. (36) reviewed experimental studies using physical aversive (painful) stimuli like heat, electric shocks or cutting to induce NSSI related responses and found that when NSSI functioned as self-punishment, NSSI individuals had a less intense pain response to painful stimuli than controls (36).

There are several models of NSSI. Favaro and Santonastaso (37) distinguish two forms of NSSI: (1) impulsive NSSI, described as an impulsive act functioning as an episodic relief after increasingly build up tension (e.g., skin cutting, burning), and (2) compulsive NSSI, described as a compulsive act expressed as a habitual, repetitive, non-functional motor behavior (e.g., hair pulling, skin picking).

Others (38) have proposed a four-factor model, which states that NSSI can be reinforced by either intrapersonal of interpersonal motives, and can be either positively reinforcing (by generating a positive feeling) or negatively reinforcing (by reducing a negative feeling). This model is supported by empirical evidence and is widely used for categorizing functionality of NSSI (29, 33). Klonsky and Muehlenkamp (29) have extended this model to seven groups of functions or motivations of NSSI: (1) affect regulation, (2) self-punishment, (3) interpersonal influence, (4) anti-dissociation, (5) anti-suicide, (6) sensation seeking, (7) interpersonal boundaries and self-control.

In summary, NSSI seems to be a maladaptive emotion regulation strategy with (internal and external) reinforcing and rewarding properties (32).

#### Neurobiology of Non-suicidal Self-Injurious Behavior

There are several neurotransmitter systems involved in NSSI. First, the endogenous opioid system, because it is involved in pain perception, pain relief and reduction of negative affect, reward and motivational processes (39, 40). Evidence for the relation between NSSI and the endogenous opioid system is based on altered endogenous opioid levels, reduced pain sensitivity and successful opioid antagonist treatment in NSSI (36, 40, 41). Risk factors for NSSI such as childhood trauma and disrupted attachment, are also related to changes in opioid levels (39, 40, 42).

The opioid system modulates the dopaminergic (DA) system (42), which is also hypothesized to be involved in NSSI. In animal models with reduced DA neurons, self-biting behavior was observed when DA-agonists were administered, whereas DA-antagonists had a reversing effect on this behavior. Self-injury in animals was mainly observed when the animal was in isolation or physically restricted (43). As DA is the major neurotransmitter involved in reward processing (44), and reward is crucial in engagement and reinforcement of NSSI, altered DA levels in the reward circuit seem to be involved in NSSI. However, humane data on the role of DA in NSSI are lacking.

Another neurotransmitter that might be implicated in NSSI is the serotonergic (5-HT) system (40). Associations have been made between 5-HT and impaired emotion regulation and impulsivity (17), which are both psychological risk factors for NSSI. Also, 5-HT dysfunction has been linked to several psychiatric disorders associated with NSSI, such as depression, anxiety, BPD and eating disorders (17, 42). Genetic studies have found a correlation between polymorphisms in 5-HT transporters and increased probability of NSSI, particularly when mediated by stress exposure (34). However, other studies found no relation between NSSI and 5-HT levels (45).

Neuroimaging studies in patients with NSSI (with or without a specific psychiatric condition) suggest involvement of brain circuits related to negative valence, reward and habit formation, and cognitive control (36). For example, NSSI-expressing individuals seem to have more inhibitory control toward NSSI-related pictures than controls. Plener et al. (46) found increased activation in the limbic system (amygdala, anterior cingulate cortex) in response toward these pictures, which was related to increased levels of arousal. They also found increased activation in the prefrontal cortex, which was related to a controlling response toward the observed limbic over-activation. This could suggest that the initial arousing response toward NSSI was neutralized, probably because of the final rewarding experience of NSSI. Kraus et al. (47) and Reitz et al. (48) found similar imaging results related to NSSI-triggering acts and while experiencing physical pain. Ammerman et al. (36) stated that these findings support the regulative effects of NSSI on emotional processing.

Liu (41) proposes a habit model to explain NSSI. The author describes a shift from voluntary behavior (instrumental learning) to repetitive habitual behavior (stimulus-response learning) occurring over time. Similar to addiction, when the self-injury becomes a habit, it loses the sensitivity to the positive outcome. This shift is paralleled by a shift from the ventral to the dorsal striatum, as observed in addiction. Other research showed that NSSI decreased arousal mostly in individuals infrequently engaging in the behavior, which suggests that the reinforcing and rewarding effects are the strongest during the onset of the behavior. The positive effects of pain relief after NSSI were lower in frequent NSSI-patients, supporting NSSI as a habitual ‘rewarding’ experience (36). This implicates that risk factors for NSSI possibly change over time, just as NSSI and its neurobiological correlates might become more habitual over time (41).

An important conclusion of both Ammerman et al. (36) and Liu (41) is that, based on most behavioral and imaging studies, NSSI is considered a conditioned, reinforcing behavioral act, aimed at emotion regulation, which results in a habitual rewarding experience. The reward system might therefore be of great interest when further investigating the neurobiological etiology of NSSI.

## Results: Psychopathological and Neurobiological Overlap Between Anorexia Nervosa and Non-Suicidal Self-Injurious Behavior

### Anorexia Nervosa and Non-suicidal Self-Injurious Behavior: Psychopathological Overlap

As mentioned above, there is a high rate of co-occurrence between EDs and NSSI. Cucchi et al. (49) found a lifetime prevalence of NSSI in 22% of the AN patients and in 33% of the bulimia nervosa (BN) patients. This seems logical because both NSSI and purging require proactive deliberate action, whereas restricting is a more passive action (50).

Claes and Muehlenkamp (51) define both NSSI and ED related behavior as “harmful behaviors falling within a behavioral spectrum ranging from self-care to self-harm.” The behavioral symptoms of EDs and more specifically of both subtypes of AN (AN-R and AN-BP) do have a highly (self-) destructive character. Because of the level of self-destructiveness in AN (and other EDs), and the assumed function of eating disorder symptoms in emotion regulation, some authors even suggest to consider EDs itself as a form of non-suicidal self-injurious behavior (NSSI) (13).

**FIGURE 1:**
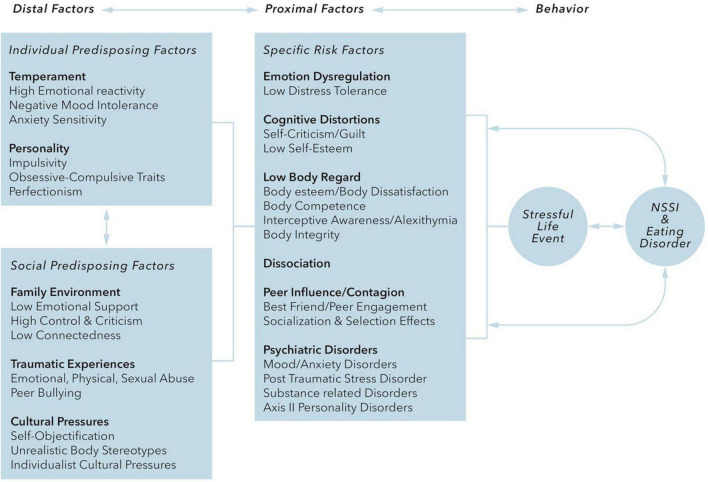
Claes and Muehlenkamp (51) distinguish “distal” risk factors that include temperament (high emotional reactivity, negative mood intolerance, anxiety sensitivity) and personality traits (impulsivity, obsessive-compulsive traits, perfectionism), cultural factors, family factors, and traumatic interpersonal experiences, and “proximal” risk factors that include emotion dysregulation, cognitive distortions, low body regard, dissociation, peer pressure/contagion and comorbid psychiatric disorders. It is hypothesized that these risk factors interact with each other and with stressful events and that the internal distress caused by these interactions is regulated by behaviors of NSSI and/or EDs, which in turn can influence or reinforce the proximal risk factors.

The onset of both AN and NSSI is usually during adolescence, and both show a trend toward a higher prevalence in the female population. Both NSSI and EDs share important risk factors like experienced childhood trauma, especially sexual abuse, or other traumatic events. Claes and Muehlenkamp present a conceptual model based on the psychosocial risk factors that are shared by NSSI and EDs (Figure 1, derived from Claes and Muehlenkamp (51).

In addition, Svirko and Hawton (13) systematically reviewed the literature on psychological associations between NSSI and EDs. The most important shared psychological factors besides affect/emotion dysregulation implicated in EDs and NSSI are impulsivity, obsessive-compulsivity, dissociation, self-criticism/self-punishment and a need for control. Furthermore, associations are found with perfectionism, body-dissatisfaction and identity-conservation (52).

Both NSSI and EDs are considered to be related to emotion dysregulation (49, 53–55). The behaviors function as maladaptive coping strategies that can be seen as a form of self-destructive behavior, to either escape or generate specific feelings. NSSI as well as eating-disordered behavior are automatically negatively reinforced by the reduction of negative affect and potentially positively reinforced by an increase in positive affect (in case of dietary restriction this could be an increase in positive mood, control and accomplishment) and are thus maintained by reinforcement and habituation. Both NSSI and EDs symptoms have been reported by patients as forms of self-punishment.

In conclusion, there seem to be psychopathological links between NSSI and AN in emotion dysregulation, (maladaptive) coping behavior, and reinforcement or reward (56).

### Anorexia Nervosa and Non-suicidal Self-Injurious Behavior: Neurobiological Overlap

The neurobiological evidence on AN and NSSI summarized above, suggests that processes related to reward are essential in their overlap. As described above, NSSI induces opioid related reward to compensate for lower levels of endogenous opioids (39, 42). If reduced sensitivity of endorphin receptors and/or low levels of endogenous opioids are typical for patients engaging in NSSI, NSSI and also anorectic/eating disordered behavior in these patients might be a way to self-stimulate the endogenous opioid system.

The endogenous opioid system and the reward system are closely linked, since opioid modulates DA pathways resulting in an increased DA release in for example the striatum. Furthermore, opioids are thought to be involved in the emotional value of reward, but also in modulating appetite and energy metabolism (17).

One class of opioids, beta-endorphin, is released during stress as well as during positive experiences and causes an euphoric rush and a reduced pain perception. NSSI, but also other impulsive behaviors like binge-eating, results in a release of beta-endorphin and its rewarding rush (39, 42, 45). Interestingly, as a consequence of malnutrition, beta-endorphin levels are reduced in AN-patients (17). NSSI, but possibly also the self-destructive eating disorder related behavior itself, in these patients could therefore be a way to self-stimulate and mobilize the last reserves of the endogenous opioid system (42). From this perspective, the state of malnutrition in AN-patients stimulates the engagement in self-destructive or other radical behavior. Besides NSSI, also binge-eating, food restriction and excessive exercising are ways to gain such an endorphin rush and thus to stimulate the reward system (39, 42, 57). In this way, self-destructive behavior is providing a short-term rewarding relief of the typical negative emotional state in AN-patients. Thereby, endogenous opioid dysfunctioning seems an important explanation for the neurobiological association between NSSI and AN.

Moreover, distilling the evidence summarized above on the neurobiology of AN and NSSI, both are linked to reward-related processes like DA dysregulation, serotonergic functioning and abnormal activation in the cortico-striatal neurocircuit.

The reward-centered model of AN (25) describing increased activation in striatal (bottom-up) networks and decreased activation in controlling cognitive (top-down) networks, is not only applicable for the engagement in anorectic behavior but also for NSSI. A study into the motivational processing of AN-compatible cues revealed that striatal DA modulation was relevant in the development of automated behavior regulated by the cortico-striatal circuit (58). This is consistent with the reward-based learning model of anorectic behavior and illness compatible cues proposed by the same authors.

Furthermore, the model of Liu (41) shows similarities with the model of O’Hara et al. (25), since both NSSI and anorectic behavior are initially a way to regulate negative affect, while repeated NSSI and AN-behavior is linked to habitual behavior or reward-associated learning. The imbalance in the cortico-striatal circuit, with a focus on bottom-up processes of the striatal reward system, might be of great relevance for the reward associated learning of self-destructive behavior in AN and NSSI in general.

Because of the central role of reward in both NSSI and AN, the reward-associated brain areas and neurobiological mechanisms, we hypothesize that reward is a central and common factor in the pathophysiology of both AN and NSSI.

## Summary

Both AN and NSSI are characterized by disturbances in affect regulation, coping strategies, the psychological constructs of reward, and on a neurobiological level dysregulation of the reward circuitry and the opioid system.

## Discussion and Conceptualization

### Anorexia Nervosa and Non-suicidal Self-Injurious Behavior: Conceptual Hypotheses

We acknowledge the fact that in this review, we have placed ourselves in the difficult position of describing links between two constructs that are conceptually and structurally dissimilar. The conceptualization of both eating disorders and NSSI is a topic of much debate. Eating disorders, and AN in particular, may be perceived as disorders of feeding, but also as body-image disorders, psychosomatic disorders, neurotic or obsessive-compulsive disorders or even disorders with a psychotic component. Based on the psychopathological and neurobiological evidence outlined in our review, a reward-related or behavioral addiction disorder perspective seems applicable.

Self-destructive behavior has long been considered a symptom of several psychiatric disorders and has only been recently taken into consideration as a separate, affect regulation- and reward-related disorder.

Based on the overlap between AN and NSSI summarized above–in epidemiology, comorbidity, clinical picture, functionality, explanatory mechanisms and involvement of (partially) the same neurobiological systems–we propose several hypothetical models of the relationship between AN and NSSI and their underlying neurobiology: (1) a nosographical approach, (2) a research domain (RDoC) approach, and (3) a network analysis approach. Central in these models is the overlap between AN and NSSI in affect regulation, body and/or pain disperception, punishment and reward.

#### Anorexia Nervosa and Non-suicidal Self-Injurious Behavior: A Nosographical Approach

**FIGURE 2:**
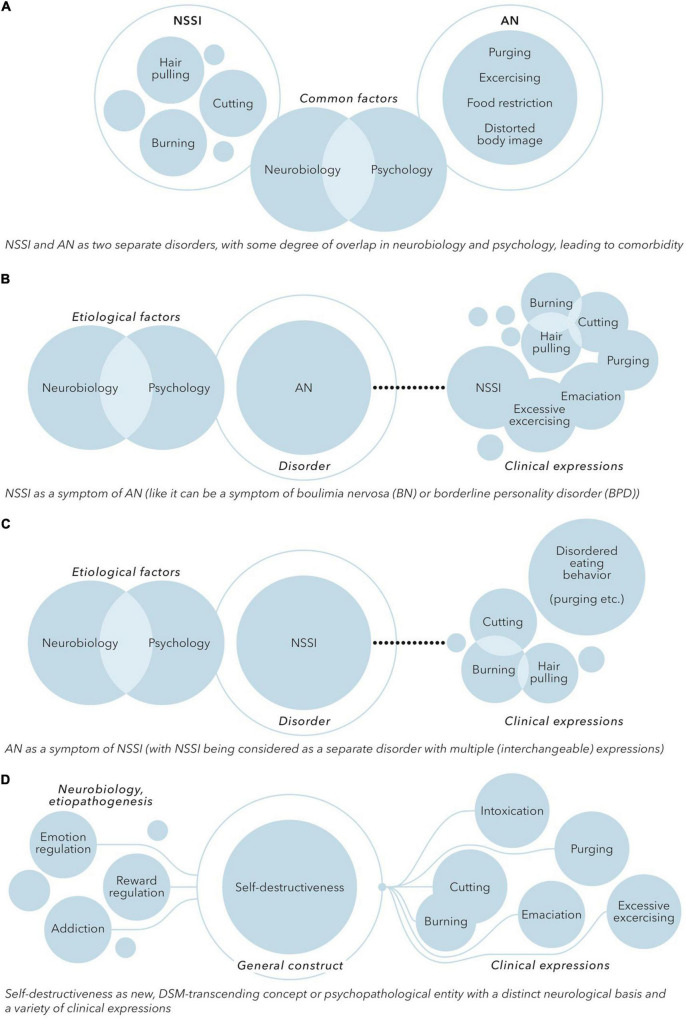
**(A)** NSSI and AN as two separate disorders, with some degree of overlap in neurobiology and psychology, leading to comorbidity. **(B)** NSSI as a symptom of AN [like it can be a symptom of boulimia nervosa (BN) or borderline personality disorder (BPD)]. **(C)** AN as a symptom of NSSI (with NSSI being considered as a separate disorder with multiple (interchangeable) expressions). **(D)** Self-destructiveness as new, DSM-transcending concept or psychopathological entity with a distinct neurological basis and a variety of clinical expressions.

Although there is considerable overlap between AN and NSSI, there are also differences. AN is a well-defined psychiatric disorder with specific diagnostic criteria. Although self-starvation, purging and over-exercising are highly self-destructive, the body image distortion and denial of the severity of the somatic condition in AN are not seen to this extent in people engaging in NSSI and seem to be specific to AN. NSSI on the other hand is increasingly being considered as a distinct disorder instead of a symptom of other disorders in the DMS-5. Based on these characteristics and differences in clusters of symptoms (i.e., a different classification of symptoms) it can therefore be hypothesized that NSSI and AN are two separate disorders, with some degree of overlap in neurobiology and functionality, leading to comorbidity (see Figure 2A).

One might also argue that NSSI is no separate construct or disorder at all. NSSI is prevalent in the context of many psychiatric disorders, especially disorders related to emotion regulation, impulsiveness, identity and interpersonal problems. Self-destructive eating disordered behavior and other forms of NSSI present during the course of AN could thus be seen as a symptom of AN, like NSSI is also often seen as a symptom in borderline personality disorder (see Figure 2B). While the initial motivation to engage in food restriction and purging might be a drive for thinness, the existing predominant view of AN as a disorder of fear of weight gain might need to be revised. In accordance with the reward-based hypothesis of AN, AN could be considered as a disorder of emotion regulation and disrupted reward processing, with self-destructiveness, reflected by eating disordered behavior as well as NSSI in general, as a key symptom.

Alternatively, yet in line with this view, the eating-disordered behavior seen in AN might be seen as a form of NSSI. In this perspective, NSSI would be considered a separate disorder with multiple (interchangeable) expressions. Purging, starvation, food restriction and over-exercising will in this model be in the same line with other forms of self-destructive behavior like for example self-cutting or self-burning. Self-destructive behavior is rewarding and reinforcing (e.g., in providing a relief of negative affect). This reinforcement is enhanced in AN due to the effects of starvation, which might explain why people engaging in eating disordered self-destructiveness tend to stick with the eating disordered behavior instead of changing to other forms of self-destructive behavior. In this model, AN, or the anorexia-related behaviors, can be considered as a form of internalized and positively reinforcing self-destructiveness or NSSI (see Figure 2C).

Based on the commonality in epidemiology, comorbidity, clinical features, functionality and neurobiological mechanisms a more fundamental relationship between NSSI and AN does not seem completely arbitrary. If a nosographical approach is conducted there seems to be considerable overlap in the intentionality and experience of both NSSI and AN. This overlap consists of the concept of self-harm, but also the concepts of aberrant reward and punishment systems, the focus on the body or bodily structures, and the concept of affect dysregulation. In this perspective, both NSSI and AN could be conceptualized as (disorders of) self-destructiveness, driven by shared factors, with a different means of expression (see Figure 2D).

#### Anorexia Nervosa and Non-suicidal Self-Injurious Behavior: A Research-Domain Centered Approach

As shown above, AN and NSSI share various psychological concepts like reward and punishment, affect regulation and control. A relatively new but promising way to investigate mental disorders is through the Research Domain Criteria (RDoC) framework (National Institute of Mental Health). The RDoC framework aims at understanding the nature of mental health and illness in terms of varying degrees of dysfunction in general psychological and biological systems (59). The RDoC framework focuses on six major domains of human functioning. These domains consist of behavioral constructs, that are studied or assessed using different classes of variables (p.e. genetic, neurocircuit, behavioral, self-report).

In our review, we have focused on the clinical and especially neurobiological overlap between AN and NSSI. The RDoC framework could be of value in further exploring the overlap between AN and NSSI in terms of exploring the basic biological and cognitive processes underlying these disorders, to explain comorbidity and to create a dimensional conceptualization of the phenomena AN and NSSI (60).

**FIGURE 3:**
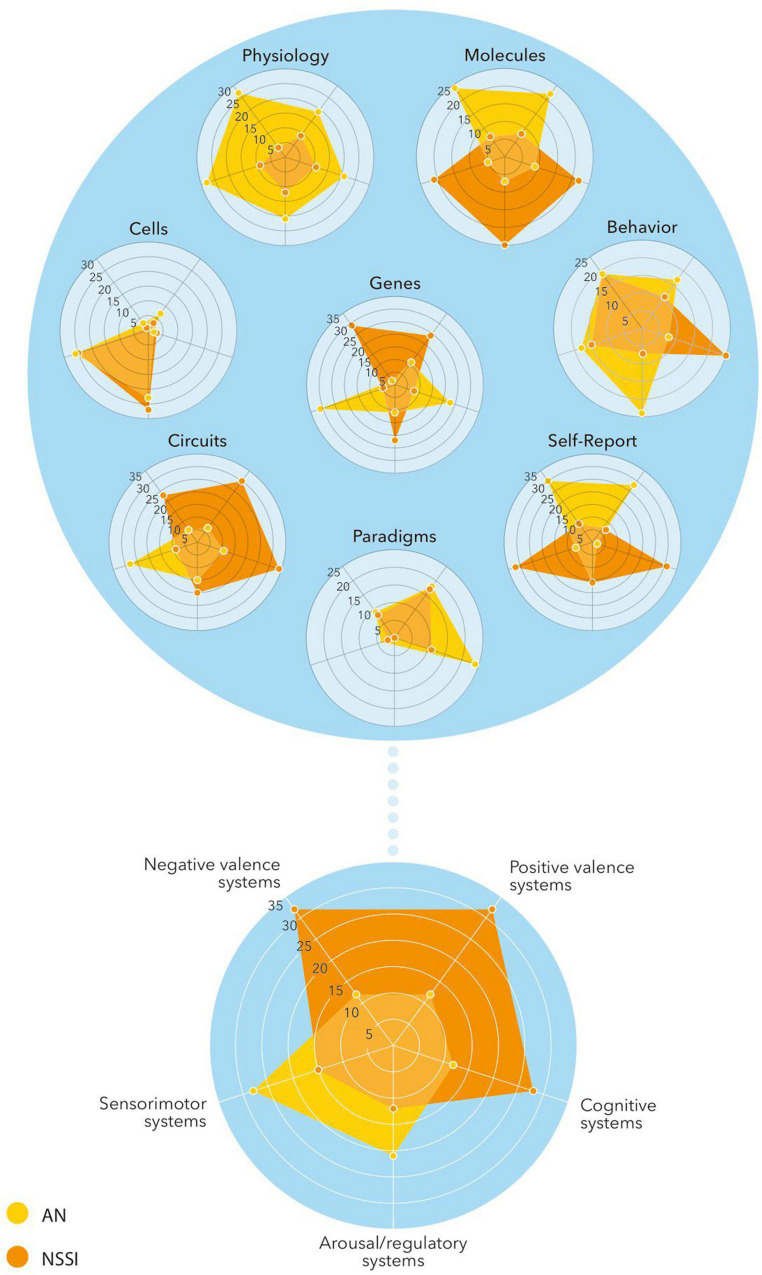
Conceptualization of the relationship between AN and NSSI *via* the RDoC framework.

According to our review, there seems to be overlap on several if not all six RDoC domains. In both disorders there is involvement of negative valence systems (e.g., fear, anxiety, non-reward), positive valence systems (p.e. reward, reward learning, habit formation), cognitive systems (p.e. self-perception, cognitive control), systems for social processes (p.e. self-perception, attachment), arousal/regulatory systems (p.e. homeostatic system, pain regulation) and sensimotor systems (motor actions, sensimotor dynamics). By using the RDoC framework it would be possible to research the more central domains and constructs of AN and NSSI, focusing on the underlying basis (and partially common) concepts. In this way the overlapping aspects can be investigated in a basic and dimensional way using genetic, neurobiological, behavioral and developmental data, leading to new (and maybe common) preventive and therapeutic options (Figure 3).

#### Anorexia Nervosa and Non-suicidal Self-Injurious Behavior: A Network Analysis Approach

Another way of researching clinical and neurobiological overlap is the network approach. In the network approach mental disorders are conceptualized as causal systems of mutually interacting symptoms (61). Statistical models provide ways to assess centrality of symptoms (i.e., how connected and clinical relevant a symptom is in a network) and to assess so-called ‘bridge symptoms,’ that occur in both disorders.

In the network approach NSSI and AN are hypothesized to co-occur due to mutual interactions among (shared) symptoms. Moreover, besides symptoms, other variables like environmental factors, developmental factors, cognitive processes and laboratory and neurobiological measurements can also be included in a network analysis. A network analysis might reveal connections and bridges between variables that seem to overlap, like the symptoms of NSSI and AN itself (the various forms of expression of NSSI as well as food restriction, binging and purging), co-occurring affective symptoms regarding mood and anxiety, but also other variables like childhood trauma, impulsivity, obsessive-compulsiveness, perfectionism, focus on the body, self-criticism, need for control, emotion regulation and reward. Based on this review it is hypothesized that maladaptive coping and emotion regulation together with dysfunctional reward processing are central variables.

**FIGURE 4:**
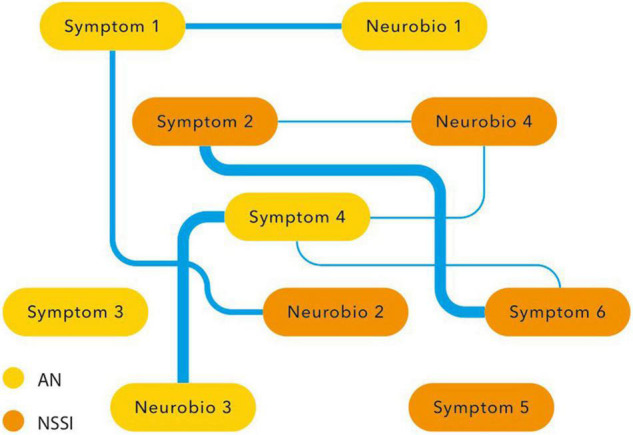
Conceptualization of the relationship between AN and NSSI *via* network analysis.

Although establishing centrality and shared symptoms might be of great value to explain co-morbidity, to predict the course of mental disorders, and to target treatment, the network analysis does not fully explain the direction and causality between connecting variables. It may, however, shed more light on the overlap between NSSI and AN and the shared etiopathogenetic and neurobiological processes underlying both phenomena. This might guide the phenomenological and research domain discussion elaborated on in the above paragraphs (Figure 4).

## Limitations

We described the (psychopathological and neurobiological) overlap between two distinct constructs: AN, which is a psychiatric diagnosis based on the internationally used psychiatric categorical classification system, and NSSI. NSSI has been included in the category ‘Conditions for Further Study’ in the DSM-5, suggesting a movement toward considering NSSI as a separate disorder. However, in the literature, NSSI is not studied as a separate disorder but as behavior occurring in patients with a range of (psychiatric) disorders as well as in patients with no disorder. Comparing two nosographically and conceptually different constructs may induce bias and complicates drawing conclusions on potential consistency. We have tried to minimize the risk of this bias by selecting literature on NSSI only, and excluding literature on self-destructive behavior and suicidality in general. Due to the use of various definitions of “self-destructive behavior” or related terms, the differences in context in which self-destructive behavior is studied, and the conceptual nature of our literature synthesis, we conducted a narrative review. This narrative review may be a prelude to more systematic review with a formal search strategy related to the theories we formulated in this narrative review.

Studies on self-destructive behavior and/or NSSI in combination with EDs often do not distinguish between the different EDs but focus on the concept of EDs in general, or on disturbed eating as a symptom. In this narrative review, we chose to focus on AN because we think that AN is a more confined illness-concept with well-defined criteria, and because we hypothesize that the anorectic behavior (restricting, purging, over-exercising) and cognitions in particular have a highly self-destructive character. We used literature specific for AN in combination with NSSI. When no information specific for AN was available, we used literature on EDs in general as this was the best available evidence on this topic.

## Conclusion

The aim of this review was to review the literature on the neurobiology of anorexia nervosa (AN), the neurobiology of non-suicidal self-injurious behavior (NSSI), and the possible clinical and neurobiological overlap between AN and NSSI, leading to a conceptual hypothesis of a shared etiopathogenesis (or etiopathological correlates) of AN and NSSI.

Based on the literature, both NSSI and EDs are related to emotion dysregulation, maladaptive coping and a dysfunctional reward system. In this conceptual review we formulate a reward-centered hypothesis explaining the overlap between AN and NSSI. Furthermore, we propose three approaches that can advance the understanding of the relationship between AN and NSSI, which integrate psychopathology and neurobiology: (1) a nosographical approach, (2) a research domain (RDoC) approach, and (3) a network analysis approach. We believe that the approaches will enhance our knowledge of the underlying neurobiological substrates and may provide groundwork for the development of new treatment options for disorders of self-destruction, like AN and NSSI.

In conclusion, we hypothesize that self-destructiveness is a new, DSM-5-transcending concept or psychopathological entity that is reward-driven, and that both AN and (other forms of) NSSI could be conceptualized as disorders of self-destructiveness.

## Recommendations for Further Research

Our models on the possible relationship between AN and self-destructiveness are hypothetical, providing new inroads for scientific substantiation. We recommend the development of research initiatives that study AN and NSSI in the context of RDoC and through the network analysis and dynamic systems approach. This might increase insight in the mechanisms that cause the clinical and neurobiological overlap between AN and NSSI. To overcome nosologic difficulties we suggest to consider NSSI as a behavioral concept independent of diagnosis, and to research the overlap and differences with disturbed eating-related behavior instead of eating disorders or anorexia nervosa. This would lead to a more neuroscientific approach of defining and researching psychopathology conform the Research Domain Criteria (RDoC) Project (59, 62).

The involvement of the opioid system in both AN and NSSI is a point of interest, possibly creating an opportunity for new pharmacotherapeutical interventions in AN, NSSI or the combination (63). For example, N-Acetylcysteine is being studied as possible moderator in behavioral addiction (64).

A potentially promising intervention that is being researched in patients with severe, chronic treatment refractory AN is modulation of the reward circuitry with deep brain stimulation (DBS). Given the observed high levels of self-destructiveness (eating-disordered as well as non-eating disordered) and the affect regulation and reward-related abnormalities in these patients, DBS might be effective in the treatment of not only AN but also of self-destructiveness in general.

Neuroimaging studies focusing on the concepts of self-destructiveness, affect regulation and reward regulation of both AN and NSSI might increase knowledge of the underlying neurobiological substrates, providing tools for the development of new and targeted treatment options for both AN, NSSI and the combination of both.

## Data Availability Statement

The original contributions presented in the study are included in the article/Supplementary Material, further inquiries can be directed to the corresponding author.

## Author Contributions

MO and JL wrote the manuscript. RM, AL, AE, and DD contributed to writing the manuscript. All authors read and approved the final manuscript.

## Conflict of Interest

The authors declare that the research was conducted in the absence of any commercial or financial relationships that could be construed as a potential conflict of interest.

## Publisher’s Note

All claims expressed in this article are solely those of the authors and do not necessarily represent those of their affiliated organizations, or those of the publisher, the editors and the reviewers. Any product that may be evaluated in this article, or claim that may be made by its manufacturer, is not guaranteed or endorsed by the publisher.

## Abbreviations

AN: Anorexia Nervosa
NSSI: Non-suicidal self-injurious behavior
RDoC: Research Domain Criteria
ED(s): Eating disorder(s)
5-HT: Serotonin
DA: Dopamine
OCD: Obsessive compulsive disorder
DSM: Diagnostic and Statistical Manual of Mental Disorders.
