# Supplementary material for: Psychopathological and Neurobiological Overlap Between Anorexia Nervosa and Self-Injurious Behavior: A Narrative Review and Conceptual Hypotheses
Source: Front Psychiatry. 2022 May 11;13:756238. doi: 10.3389/fpsyt.2022.756238 (PMC9130491; doi:10.3389/fpsyt.2022.756238)
Supplement: Supplementary file 1 [file Table_1.DOCX]

| **Neural circuits** | **Brain areas** | **Functionality** | **fMRI results in AN*** | **Hypothesis in AN** |
| --- | --- | --- | --- | --- |
| Default mode network (DMN) | Posterior cingulate cortex  Medial prefrontal cortex  Medial temporal cortex  Inferior parietal cortex | Interoception  Self-relevant mentalizing | ↑ DMN connectivity  ↑ activation dorsal posterior cingulate cortex  ↓ activation parietal cortices | Inability to come to an internal restful state  ↓ visuospatial bodyprocessing  Distortion and avoidance of body-related information |
| Salience and reward networks (SN) | Insula (primary gustatory cortex)  Anterior cingulate cortex (ACC)  Oribitofrontal cortex (OFC)  Ventral striatum (VS) | Processing of taste, incentive value of food  Assessment and integration of motivated/reward behavior  Processing of emotional tone  Interoception  Proprioception  Error monitoring  reward expectation  Reward valuation  Motivation  Reward approach | ↓/↑ activation insula (cue dependent)  ↑ SN connectivity  ↓/↑ activation ACC  ↑ functional connectivity  ↑ activation striatum | Experience of a generally aversive interoceptive state  Alterations in perception of reward  Diminished response to taste and to the rewarding value of foods  Aversive perceptions of food  ↑ salience attribution to rewarding and aversive food stimuli? (↑ sensitivity to food stimuli, irrespective of valence)  ↓readiness to approach food  More efficient control circuitry  Reward contamination  Reward contamination  ↑ sensitivity to punishment?  ↑ sensitivity to disorder-relevant stimuli  ↑ attribution of motivational salience towards illness-related cues |
| Executive control network (ECN) | Prefrontal cortex (PFC) | Executive control  Decision making  Emotion regulation | ↑/↓ connectivity (disbalance)  ↑ activation medial PF  ↑ activation dorsolateral PFC | ↑ cognitively mediated food avoidance |

**Supplement 1**. Findings in fMRI studies in AN according to Frank e.a. (2019) and O’Hara e.a. (2015) and the hypotheses regarding the significance of these findings in the context of AN.

In analogy with Frank e.a. (2019) one may distinguish a default network (DFN, involved in interoception and self-relevant metallization), salience (SN), a reward network (including the limbic circuit and involved in orienting subjects to support food approach, decision-making and reward-processing), and an executive network (ECN, implicated in executive control, long-term decision making and emotion regulation). Dysfunctional activation in the orbitofrontal cortex (OFC), also related to reward processing and especially reward valuation, is thought to be implicated in the concept of ‘reward contamination’, in which AN patients seem to experience normal food cues as punishing and illness compatible cues as rewarding.
